# Effect of mobile phone-based health insurance contribution payment system on retention of coverage in the National Health Insurance Scheme in Ghana: an evaluation study

**DOI:** 10.1186/s12913-023-09236-7

**Published:** 2023-03-11

**Authors:** Eric Nsiah-Boateng, Mariam Musah, Collins Danso Akuamoah, Francis Asenso-Boadi, Francis-Xavier Andoh-Adjei, Bernard Okoe Boye

**Affiliations:** 1 National Health Insurance Authority, Accra, Ghana; 2 Ministry of Health, Accra, Ghana; 3 School of Public Health, University of Ghana, Accra, Ghana

**Keywords:** Impact evaluation, Digital health, Health insurance, Retention of coverage, Ghana

## Abstract

**Background:**

Ghana introduced a mobile phone-based contribution payment system in its national health insurance scheme (NHIS) in December 2018 to improve the process of enrolment. We evaluated the effect of this digital health intervention on retention of coverage in the Scheme, one year after its implementation.

**Methods:**

We used NHIS enrolment data for the period, 1 December 2018–31 December 2019. Descriptive statistics and propensity-score matching method were performed to examine a sample of 57,993 members’ data.

**Results:**

Proportion of members who renewed their membership in the NHIS via the mobile phone-based contribution payment system increased from 0% to 8.5% whilst those who did so through the office-based system only grew from 4.7% to 6.4% over the study period. The chance of renewing membership was higher by 17.4 percentage points for users of the mobile phone-based contribution payment system, compared to those who used the office-based contribution payment system. The effect was greater for the informal sector workers, males and the unmarried.

**Conclusions:**

The mobile phone-based health insurance renewal system is improving coverage in the NHIS particularly for members who hitherto were less likely to renew their membership. Policy makers need to devise an innovative way for new members and all member categories to enrol using this payment system to accelerate progress towards attainment of universal health coverage. Further study needs to be conducted using mixed-method design with inclusion of more variables.

**Supplementary Information:**

The online version contains supplementary material available at 10.1186/s12913-023-09236-7.

## Background

Many Low- and Middle-Income Countries (LMICs) are implementing digital health innovations in their health systems towards achieving universal health coverage (UHC) [1–3], a fundamental principle of Sustainable Development Goal (SDG) 3.8. Ghana started its UHC journey in 2003 with the establishment of a National Health Insurance Scheme (NHIS). Prior to this, healthcare was financed strictly from out-of-pocket (OOP) payment at the point of service use following a transition from free healthcare at independence in 1957 to the introduction of user fees in the 1980s [4]. The OOP payment impoverished an already poor population and consequently excluded a large majority of the people from accessing care [5, 6]. It also reduced utilization of healthcare services and led to deteriorated health outcomes. There was no protection for the poor and healthcare financing was highly inequitable. The introduction of the NHIS; therefore, removed this financial barrier and allowed people to access care without paying at the point of use [7, 8].

The NHIS provides annual healthcare cover for its members at a defined contribution of GHS22.00 (US$3.78)^1^ and/or processing fee [9] of GHS8.00 (US$1.38) for new members and GHS5.00 (US$0.86) for existing members. Membership is mandatory by law but voluntary in practice due to lack of enforcement. Members are broadly categorized into exempt and non-exempt group. The exempt group pays no contribution to the scheme, and they include: 1) persons below the age of 18 years: 2) indigent; 3) Social Security and National Insurance Trust (SSNIT) pensioners; 4) persons aged 70 years or older; 5) pregnant women; and 6) beneficiaries of the Livelihood Empowerment Programme against Poverty (LEAP). The non-exempt group, however, pays contribution directly to the scheme and comprises workers in the informal sector, including the self-employed. Membership categories are dynamic, in that a member may change from one category to another over a period.

The NHIS relies heavily on government funding through an earmarked fund, the National Health Insurance Fund (NHIF). This constitutes about 74% of the total funding of the NHIS. The NHIF is funded through a 2.5% value-added tax (VAT) on selected goods and services. The remaining funding comes from informal sector workers’ contributions; ﻿two- and one-half percentage points of each person’s contribution to the SSNIT; return on investments; and sector budgetary support from government [9]. The NHIS offers a comprehensive healthcare cover for outpatient and inpatient services, including investigations and medications on the NHIS medicines list. The benefit package reportedly covers about 95% of disease conditions in Ghana [10]. There are over 4000 public and private healthcare facilities credentialed to provide services for members of the Scheme.

The NHIS has improved population coverage and access to healthcare services utilization since its introduction almost two decades ago [7, 8]. However, over the 2016–2018 period, the scheme recorded consistent decline in population coverage after reaching a peak of 11.3 million members (40%) in 2015. For instance, 10.8 million people, representing 36% of the population were active members of the scheme in 2018 [11]. This was due to the inconvenience associated with the registration process at the district offices of the Scheme. Many people experienced long queues and hours of waiting time to enrol in scheme. Distance to a district office, and time and travel cost were also found as barriers to enrolling and staying enrolled in the NHIS [12–14]. In response, Management of the scheme implemented a mobile phone-based contribution payment system in December 2018 to provide an easy, convenient, and affordable way for members to renew their membership annually without being physically present at an NHIS branch office.

Our review of the literature showed paucity of studies on evaluation of digital health intervention particularly in the health insurance industry, indicating a gap in knowledge in this area of study. A prospective study on the use of the NHIS mobile phone renewal system conducted in one geographic region of Ghana focused on determinants of renewing membership in the scheme through the mobile phone payment system [15]. The study found that factors such as living in urban centres; higher level of education; informal sector employees; and paying premium using the mobile phone were associated with membership renewal in the Scheme [15]. A cross-sectional survey in Kenya also found that mobile phone use increased the probability of enrolling in the National Health Insurance Fund [2]. However, an intervention study on the effect of paying health insurance subscription using mobile money (M-Pesa) in Kenya found no significant effect on enrolment [3]. Given that Ghana’s mobile phone-based contribution payment system is a new digital health solution, and there is little knowledge on its effect on enrolment, we sought to evaluate effect of this intervention on retention of coverage for informed decision-making on its modification and scale-up.

### The NHIS mobile phone-based contribution payment system

The NHIS mobile phone-based contribution payment system, popularly referred to as “mobile renewal” is a two-part system operating two distinct processes: 1) a mobile-based renewal process; and 2) a non-biometric authentication process. While the former is used by members to renew their membership in the scheme, the latter is used at healthcare facilities by providers to authenticate attendance of NHIS members and provide feedback in the form of text message to the purchaser, the National Health Insurance Authority (NHIA). The mobile phone-based contribution payment renewal process is based on a simple Unstructured Supplementary Service Data (USSD) application integrated onto the existing mobile money platform. It begins with an SMS reminder to members, notifying them of their membership expiration dates. Two reminders are sent: 1) one month prior to expiration; and 2) two weeks prior to expiration.

The renewal process starts by dialing a dedicated USSD short code (*929#), available across the three major telecommunication networks in the country: MTN, Vodafone and Airtel-Tigo. The first step requires members to input their membership number on their membership cards. Once this is done, the renewal system interacts with the NHIS membership database to determine membership category and the corresponding contribution to be paid. The contribution due depends on the last renewal location (district). This is due to variations in contributions across regions. A member then follows the prompts thereafter to complete the renewal process. Once renewal is successful, a confirmation message is sent to the member.

In addition to the renewal process, the interface also allows a member to access information on the benefits package and the NHIS medicines list. The NHIA charges a GHS1.00 (US$0.17) convenience fee for the use of the mobile phone-based contribution payment system. This is added to the contribution before payment is completed. The mobile phone-based contribution payment system is available to all member categories except pregnant women and indigents (core poor). These two categories still need to go to the district offices to renew their membership in the scheme.

The second part of the mobile phone-based contribution payment system begins when a member visits a health facility to access care. The provider also dials a dedicated short code (*842#) using a pre-registered telephone number. Using the unique member number on the membership card, the provider confirms validity of the card. Once validity is established, a numerical code, dubbed the Claims Check Code (CCC) is sent to the provider. This code is captured on the claims form to validate a member’s attendance at the facility. Thus, claims submitted without CCC are rejected. Again, at the time of CCC generation, a Short Message Service (SMS) is sent to the member as confirmation of attendance. The SMS provides an opportunity for the member to contact the NHIS if they were not at the facility. This serves as a feedback mechanism which enables the NHIS to gather data on attendances which may not be valid.

## Methods

### Study design and sampling

The study is a cross-sectional evaluation study of NHIS mobile phone-based contribution payment system, which was implemented in December 2018 to provide convenience for members of the scheme to renew their membership. We sourced enrolment data from NHIA, covering the period 1 December 2018–31 December 2019. We randomly sampled a total of 57, 993 out of 21 million enrolment data of members who had renewed their membership, either through the mobile phone-based contribution payment system or the in-person BMS system at the NHIS branch offices nationwide.

Characteristics of the sampled enrolment data were NHIS member number, type of registration (new or renewal), and sex. Other characteristics included age, defined NHIS member categories such as informal sector workers, indigents, and SSNIT contributors, etc.; and platform for registration, which is either through the office-based biometric management system or the mobile phone-based system.

### Data analysis

We performed descriptive analysis to explore socio-demographic characteristics and trends in membership renewal of the sampled population by type of enrolment payment system (in-person biometric enrolment system versus mobile phone-based renewal payment system). We also employed multivariate logistic regression analysis to estimate the odds of a member retaining coverage in the scheme.

Moreover, we applied treatment-effects estimation method using the logic specification of the propensity score matching to (PMS) evaluate effect of the intervention on membership renewal. The use of PSM technique for this study is based on the observational data employed and the fact that there was no baseline. The PMS is useful for the selection of comparison/control group *ex post* from reasonably large administrative data through a matching procedure based on observed characteristics [16–18].

We estimated the probabilities of renewing membership using the mobile phone-based payment system (propensity scores) for each member of the treatment and control groups based on similarities in observed characteristics, including sex, age, marital status, and membership category. This technique assumes no selection bias based on unobserved characteristics of the two groups [16, 17, 19]. We then matched the control group to the treatment group based on the propensity scores, that is, the estimated probabilities of being treated (or renewing membership in the NHIS using the mobile phone-based payment system). This analytical technique is recognized for its ability to remove biases generated through differences in observational characteristics and increase balance between treatment and control groups [19, 20]. It has been used in other studies in Ghana [21, 22] and elsewhere [23, 24] to evaluate effect of social protection programmes.

**Fig. 1:**
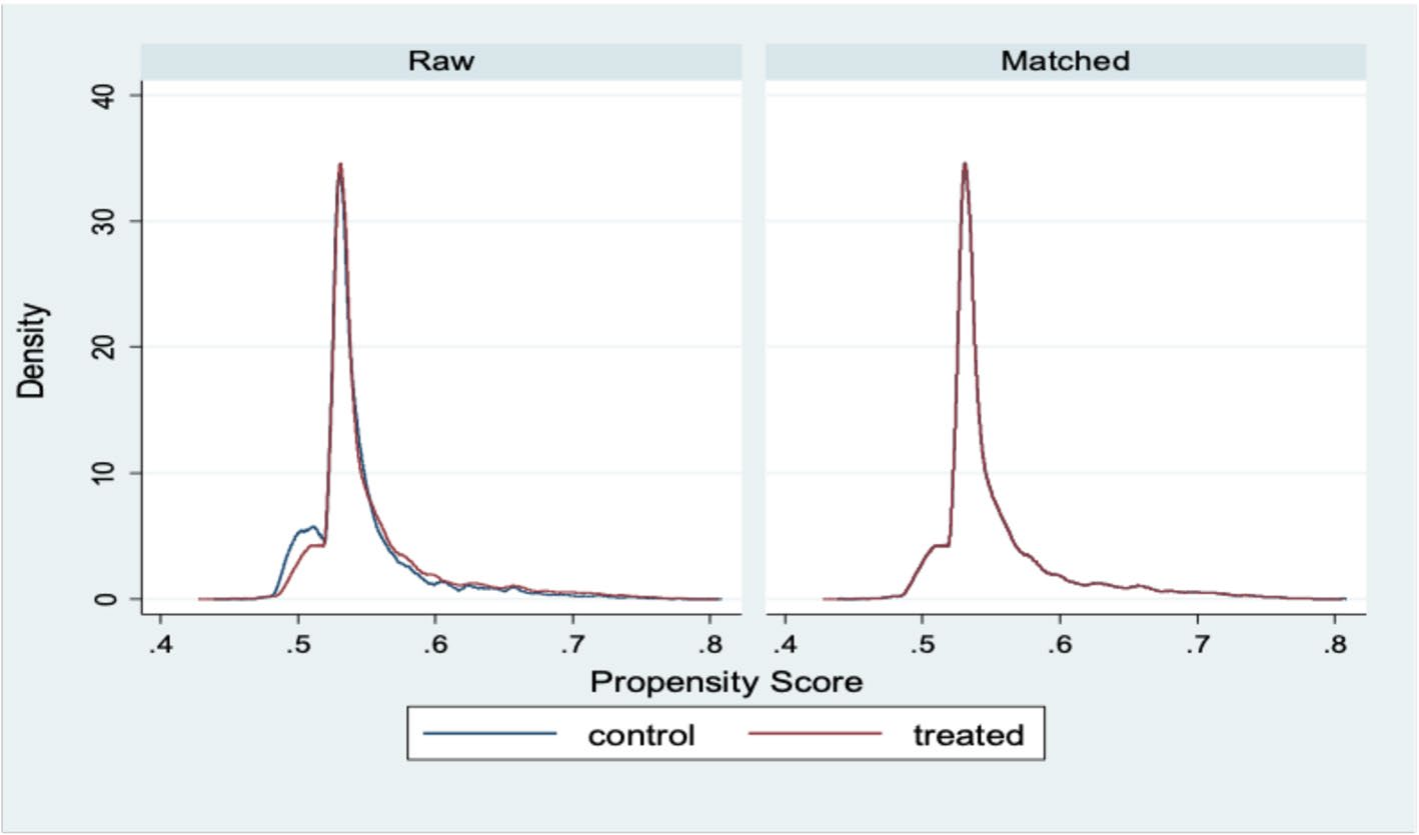
Balance plot of propensity scores for control and treatment groups

Members who renewed their membership in the scheme through the mobile phone-based payment system were the treatment group, assigned a value of “1” and those who used the in-person biometric payment system were the control group, assigned a value of “0”. The explanatory variables were sex, age, marital status, and membership category, broadly categorised into premium contributors (non-exempt group) and non- premium contributors (exempt group). The choice of these variables was driven by the NHIS enrolment data. A balance test for the intervention and control groups was performed using these explanatory variables and the results showed that the two groups were balanced (Fig. 1).

We also performed sensitivity analysis using other treatment effects estimation methods such as inverse-probability weights (IPW), IPW regression adjustments, and nearest-neighbour matching (NNM) to test robustness of our findings (Supplementary Table 1). Microsoft excel and STATA version 14 were used to analyse the data.

## Results

### Characteristics of the sampled population

Approximately, 80.2% of the members renewed their membership in the NHIS overall (Table 1). Average age of the members was 24.9 years (*SD* = 21.46); 59.1% were females; 62.5% had never married (single); and 34% were informal sector workers (non-exempt group). Except for marital status, there were significant differences in socio-demographic characteristics between members who used the office-based payment system and those who used the mobile phone-based payment system to renew their membership in the scheme (not shown in Table 1). These differences, however, were addressed by using the propensity score matching method to estimate and match probabilities of renewing membership in the NHIS through the mobile phone-based payment system for each member of the two groups based on similarities in their observed characteristics.

**Table 1:** Characteristics of the control and treatment group before matching (*n* = 57,993)

Variable	Type of contribution payment system		Total
	Office-based payment system (Control)	Mobile phone-based payment system (Treatment)	
**Registration type**			
New	11,467 (39.7)	0^b^	11,467 (19.8)
Renewal	17,427 (60.3)	29,099 (100.0)	46,526 (80.2)
**Sex**			
Female	16,857 (58.3)	17,394 (59.8)	34,251 (59.1)
Male	12,037 (41.7)	11,705 (40.2)	23,742 (40.9)
**Age (Mean, Std.)**	23.5(20.3)	26.3 (22.5)	24.9 (21.5)
**Marital status**^**a**^			
Divorced	64 (0.5)	96 (0.6)	160 (0.6)
Married	4,923 (37.9)	5,870 (37.2)	10,793 (37.5)
Single	7,826 (60.3)	9,507 (60.2)	17,333 (60.2)
Widowed	177 (1.4)	322 (2.0)	499 (1.7)
**Membership category**			
Exempt group	20,542 (71.1)	17,710 (60.9)	38,252 (66.0)
Non-exempt group	8,352 (28.9)	11,389 (39.1)	19,741 (34.0)

^a^Valid response is 28,785

^b^The mobile phone-based contribution payment system was being used for renewal of membership only at time of the study

### Trends in coverage retention

**Fig. 2:**
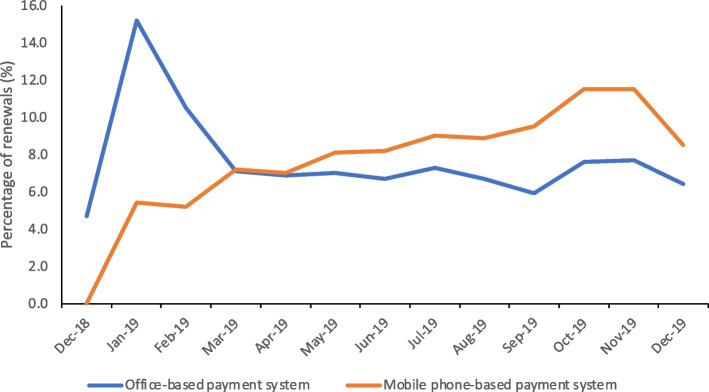
Trends in membership renewal by type of enrolment payment system, December 2018-December 2019

The proportion of members who renewed their membership over the study period through the mobile phone-based contribution payment system increased consistently from 0% to 8.5% (Fig. 2). Those who renewed at the NHIS office, however, went up sharply between the first two months from 4.7% to 15.2% and declined thereafter to 6.4%.

### Factors associated with membership renewal in the NHIS

The multivariate logistic regression model showed that the unmarried (OR = 1.75, 95% CI: 1.54–1.98) and older adults (OR = 1.04, 95% CI: 1.03–1.04) were significantly more likely to retain coverage in the scheme than the married and young adults, respectively (Table 2). However, the males (OR = 0.69, 95% CI: 0.64–0.75) were significantly less likely to retain coverage in the scheme compared to the females. Likewise, the non-exempt groups (informal sector workers) (OR = 0.70, 95% CI: 0.63–0.77) were less likely to retain coverage relative to those exempted from paying contribution to the scheme.

**Table 2:** Multivariate logistic regression estimates for retention of coverage

Variable	OR	Std. Err	[95% C. I]	
Female	Ref			
Male	0.69***	0.028	0.64	0.75
Age	1.04***	0.002	1.03	1.04
Married	Ref			
Unmarried	1.75***	0.110	1.54	1.98
Exempt group (non-contributors)	Ref			
Non-exempt group (contributors)	0.70***	0.036	0.63	0.77
_cons	3.05***	0.236	2.63	3.55
Number of obs = 28,785				
LR chi2(4) = 845.23				
Prob > chi2 = 0.0000				
Log likelihood = -8995.1764				
Pseudo R2 = 0.0449				

*OR* Odds Ratio, *C.I* Confidence Interval

^***^p < 0.001

### Effects of mobile phone-based contribution payment system on coverage retention

The propensity-score matching estimates showed significant effect of the mobile phone-based contribution payment system on membership renewal in the NHIS (Table 3). Overall, the chance of renewing membership in the scheme was higher by 17.4 percentage points for members who used the mobile phone-based application system than those who used the conventional in-person biometric membership system (BMS) at the district offices. In addition, the probability of renewing coverage in the scheme was higher by 21.3 percentage points for the informal sector workers (premium payers) who used the mobile phone-based application system than those who used the BMS. Similarly, the probability of renewing membership in the scheme was higher by 20.9 percentage points for the males who used the mobile phone-based contribution payment system than those who used the BMS.

**Table 3:** Average treatment effect (ATET) estimates for renewal of membership (*n* = 28,785)

Variable	Coef	AI Robust Std. Err	[95% C.I]	
Overall	0.174***	0.004	0.167	0.181
Male	0.209***	0.006	0.197	0.221
Female	0.151***	0.004	0.143	0.159
Married	0.147***	0.005	0.137	0.158
Unmarried	0.189***	0.005	0.180	0.198
Exempt group	0.162***	0.005	0.153	0.171
Non-exempt group	0.213***	0.007	0.200	0.226

^***^p < 0.001

*ATET* Average treatment effect on the treated, *C.I* Confidence Interval

### Sensitivity and robustness test

Results of the other treatment effects estimation methods demonstrated consistency in the positive impact of the mobile phone-based payment system on membership renewal in the NHIS. Two estimation methods (inverse-probability weights and inverse-probability weights regression adjustments), however, had a higher ATET than the nearest-neighbour matching method (21.5 percentage points vs 17.4 percentage points) (Supplementary Table 1). Results of the subgroup analysis also showed that the effect was more pronounced for the informal sector workers, males and the unmarried.

## Discussion

This study evaluated effect of the digital health intervention implemented in the NHIS to speed up progress of enrolment towards attainment of UHC. It also examined factors influencing members’ decision to renew their memberships in the scheme. The findings reveal that the mobile phone-based contribution payment system increases retention of coverage in the NHIS overall. As expected, the informal sector workers, the males and the unmarried who hitherto were less likely to enrol in the NHIS, are renewing their coverage in the scheme through the newly implemented mobile phone-based contribution payment system more than those using the in-person biometric enrolment payment system at the district offices.

The possible explanation for the positive impact of the mobile phone-based contribution payment system is that it removes barriers to membership renewal, including time and travel cost, as found in other studies [12, 13]. Prior to the introduction of this digital health intervention, individuals had to queue for long hours at the district offices to either enrol as new members or renew their membership in the NHIS [12, 13], and others experienced delays in the processing of their cards [14, 25]. Consequently, these barriers discouraged them to participate in the scheme [14]. Our findings corroborate studies in other development areas where the use of mobile phone and mobile phone-based payments showed positive impact on farm productivity [26], poverty reduction and economic growth [27]. Findings of this present study, however, contradicts one in Kenya [3], where payment of premium through mobile phone yielded no significant effect on enrolment in the National Health Insurance Fund. The contradiction might be due to the randomized experiment design employed in the Kenyan study.

Although the mobile phone-based contribution payment system is improving enrolment of males and informal sector workers in the NHIS, these groups are less likely to renew membership in the scheme overall, compared to their respective counterparts. The feeling of perceived good health (rare illness) and poor quality of services at healthcare provider sites, as found in the other studies [14, 25, 28, 29], could be the underlying reasons for this revelation. Our findings are consistent with an earlier study on the NHIS [29] but contradict another study that was conducted in one district of the Greater Accra region [30], where the males and informal sector workers were more likely to retain coverage in the scheme. Difference in the findings might be due to scope of the two studies with respect to the study area and population.

Findings of this study, however, show that the older adults of the scheme are more likely to renew their membership, probably due to their higher healthcare needs compared to the younger adults. This finding confirms an earlier study [30] but contradicts a study by Van der et al. [31], which examined determinants of enrolment in the NHIS using datasets from Global Ageing and Adult Health (2007–2008) and Ghana Living Standards Survey round six (2012–2013). Surprisingly, the unmarried are also more likely to retain coverage in the scheme compared to the married. This finding contradicts an earlier study [29], where the married individuals were rather more likely to retain coverage in the scheme compared to the single individuals. The difference in the findings is due to the operational definition adopted. In this present study, the unmarried comprised the single, divorced and widowed whilst the other study operationalized single as the “never married” individuals.

Our findings suggest that leveraging electronic-based payment systems such as mobile phone-based payments (mobile money), can remove barriers (time and travel cost) to enrolment and speedy up progress towards realization of UHC by 2030. Besides, the decreased likelihood of the informal sector workers and the males renewing their memberships indicates that system-wide factors other than time and travel costs, for example, perceived poor quality of service and OOP, reported in earlier studies [14, 25, 28, 29] are important determinants of enrolment, which policy makers need to address to improve enrolment in the scheme.

### Limitations

We encountered few limitations in the study. First, the mobile phone-based contribution payment system has only one level (membership renewal). It could not be used by persons who wished to enrol in the Scheme for the first time. This situation might over-estimate the treatment effect. However, the matched sample was balanced, and results of the sensitivity analysis were also consistent, indicating that the findings are robust for policy decision making. Secondly, the one-level design limitation made it impossible to separately examine factors influencing members’ decision to renew their memberships using this system and the conventional biometric system at the district offices. Nonetheless, the overall logistic model specification used, provides insight into associated factors of retention of coverage in the NHIS.

Moreover, the NHIS membership database had fewer explanatory variables, which accounted for less than 5% of the variations in the renewal of membership. The limited variables also made the estimation of the ATET prone to unobservable bias. Characteristics including education and household size, which are found to be associated with enrolment in health insurance were unavailable in the enrolment dataset. Nonetheless, the large sample size used for the study, addresses this limitation.

Lastly, the PSM technique helps to address selection bias on the observables but not omission of unobserved differences between the treatment and the control groups (endogeneity). Therefore, our findings rely on the assumption that there was no selection bias based on unobserved characteristics of the two groups within the one-year period of the study.

## Conclusions

The mobile phone-based contribution payment system is improving retention of coverage in the NHIS particularly groups who hitherto were less likely to renew their membership in the scheme (males, informal sector workers, and the unmarried). Management of the scheme needs to speedy up the process of making the system possible for the population to enrol as new members to improve enrolment in the NHIS towards UHC. A mixed-method study with inclusion of more variables would also be necessary to understand why these groups have lower chance of renewing their membership in the NHIS despite introduction of digital health intervention, which addresses time and travel costs associated with participation in the scheme.

## Supplementary Information

**Additional file 1: Supplementary Table 1.** ATET robustness test for coverage retention.

## Data Availability

The data for this study is publicly available in Mendeley Data Repository with the Reserved https://doi.org/10.17632/myfbx9g4tg.1, https://data.mendeley.com/datasets/myfbx9g4tg

## Abbreviations

ATET: Average Treatment Effect on the Treated
BMS: Biometric Membership System
CCC: Claims Check Code
GHS: Ghanaian Cedis
ILO: International Labour Organisation
IPW: Inverse-probability weights
LEAP: Livelihood Empowerment Programme against Poverty
LMICs: Low and Middle-Income Countries
NHIA: National Health Insurance Authority
NHIF: National Health Insurance Fund
NHIS: National Health Insurance Scheme
NNM: Nearest-neighbour matching
OOP: Out-of-Pocket Payment
PSM: Propensity Score Matching
SDG: Sustainable Development Goals
SMS: Short Message Service
SSNIT: Social Security and National Insurance Trust
UHC: Universal Health Coverage
USSD: Unstructured Supplementary Service Data
VAT: Value-Added Tax

## Footnotes

1 Exchange rate for October 21, 2020: US$1.00 = GHS5.81.
