# Supplementary material for: Effect of mobile phone-based health insurance contribution payment system on retention of coverage in the National Health Insurance Scheme in Ghana: an evaluation study
Source: BMC Health Serv Res. 2023 Mar 11;23:239. doi: 10.1186/s12913-023-09236-7 (PMC10007802; doi:10.1186/s12913-023-09236-7)
Supplement: Supplementary file 1 — Additional file 1: Supplementary Table 1. ATET robustness test for coverage retention. [file 12913_2023_9236_MOESM1_ESM.docx]

**Supplementary Table 1. ATET robustness test for coverage retention**

| **Variable** | **Coef.** | **Robust**  **Std. Err.** | **P>z** | **[95% C.I]** | |
| --- | --- | --- | --- | --- | --- |
| **Inverse-probability weights (IPW)** |  |  |  |  |  |
| Overall | 0.214 | 0.004 | 0.000 | 0.207 | 0.221 |
| male | 0.274 | 0.006 | 0.000 | 0.262 | 0.286 |
| female | 0.177 | 0.004 | 0.000 | 0.169 | 0.185 |
| married | 0.149 | 0.005 | 0.000 | 0.139 | 0.159 |
| unmarried | 0.252 | 0.005 | 0.000 | 0.242 | 0.261 |
| exempt group | 0.242 | 0.005 | 0.000 | 0.231 | 0.252 |
| non-exempt group (informal sector worker) | 0.218 | 0.006 | 0.000 | 0.206 | 0.231 |
| **IPW regression adjustment** |  |  |  |  |  |
| Overall | 0.214 | 0.004 | 0.000 | 0.207 | 0.221 |
| male | 0.274 | 0.006 | 0.000 | 0.262 | 0.287 |
| female | 0.177 | 0.004 | 0.000 | 0.169 | 0.186 |
| married | 0.149 | 0.005 | 0.000 | 0.139 | 0.159 |
| unmarried | 0.252 | 0.005 | 0.000 | 0.242 | 0.261 |
| exempt group | 0.239 | 0.005 | 0.000 | 0.229 | 0.248 |
| non-exempt group (informal sector worker) | 0.219 | 0.006 | 0.000 | 0.207 | 0.231 |
| **Nearest-neighbour matching** |  |  |  |  |  |
| Overall | 0.173 | 0.003***^a^*** | 0.000 | 0.167 | 0.180 |
| male | 0.210 | 0.006***^a^*** | 0.000 | 0.198 | 0.222 |
| female | 0.151 | 0.004***^a^*** | 0.000 | 0.142 | 0.159 |
| married | 0.146 | 0.005***^a^*** | 0.000 | 0.136 | 0.157 |
| unmarried | 0.189 | 0.005***^a^*** | 0.000 | 0.180 | 0.198 |
| exempt group | 0.161 | 0.005***^a^*** | 0.000 | 0.152 | 0.171 |
| non-exempt group (informal sector worker) | 0.213 | 0.007***^a^*** | 0.000 | 0.200 | 0.226 |

**Note:**

***^a^*AI Robust Std. Err**
